# Fluorescence Polarization Switching from a Single Silicon Vacancy Colour Centre in Diamond

**DOI:** 10.1038/srep12244

**Published:** 2015-07-23

**Authors:** Yan Liu, Gengxu Chen, Youying Rong, Liam Paul McGuinness, Fedor Jelezko, Syuto Tamura, Takashi Tanii, Tokuyuki Teraji, Shinobu Onoda, Takeshi Ohshima, Junichi Isoya, Takahiro Shinada, E Wu, Heping Zeng

**Affiliations:** 1 State Key Laboratory of Precision Spectroscopy, East China Normal University, Shanghai 200062, P. R. China; 2 Institute for Quantum Optics, University Ulm, Albert-Einstein-Allee 11, 89081 Ulm, Germany; 3 School of Fundamental Science and Engineering, Waseda University, 3-4-1 Ohkubo, Shinjuku, Tokyo 169-8555, Japan; 4 National Institute for Materials Science, 1-1 Namiki, 304-0044 Tsukuba, Japan; 5 Japan Atomic Energy Agency, 1233 Watanuki, Takasaki, Gunma 370-1292, Japan; 6 Research Centre for Knowledge Communities, University of Tsukuba, 1-2 Kasuga, Tsukuba, Ibaraki 305-8550, Japan; 7 Centre for Innovative Integrated Electronic Systems (CIES), Tohoku University, 468-1 Aramaki Aza Aoba, Aoba-ku, Sendai, Miyagi, 980-0845, Japan

## Abstract

Single-photon emitters with stable and uniform photoluminescence properties are important for quantum technology. However, in many cases, colour centres in diamond exhibit spectral diffusion and photoluminescence intensity fluctuation. It is therefore essential to investigate the dynamics of colour centres at the single defect level in order to enable the on-demand manipulation and improved applications in quantum technology. Here we report the polarization switching, intensity jumps and spectral shifting observed on a negatively charged single silicon-vacancy colour centre in diamond. The observed phenomena elucidate the single emitter dynamics induced by photoionization of nearby electron donors in the diamond.

Diamond is a beautiful and precious stone, but it is the defects in the diamond crystal that attract much attention for research around the world. Some of the colour centres in diamond are bright and with good photoluminescence (PL) stability at room temperature^1^,^2^,^3^,^4^,^5^,^6^. The nitrogen vacancy (NV) colour centre is one of the most studied diamond defects. The optical properties of the NV centre in diamond are conducive to many applications, such as bio-markers for living cells due to the nontoxic diamond host, practical single-photon sources for quantum cryptography, demonstrations of fundamental physics in quantum optics, and sensitive nanoscale sensors of temperature as well as electric and magnetic fields^7^,^8^,^9^,^10^,^11^,^12^,^13^. Lately, the negatively charged silicon vacancy (SiV^−^) colour centre has been reported as a good candidate for single-photon source due to its remarkable optical properties and electronic structure^14^,^15^. At room temperature, the PL from SiV colour centres can show near 100% polarization contrast, and therefore have high potential for applications in quantum information processing and cryptography^16^. SiV^−^ centres also possess advantages for optical quantum information applications due to their narrow zero phonon line (ZPL) at 738 nm with high fluorescence concentration and short fluorescence lifetime^17^,^18^,^19^,^20^,^21^,^22^.

Polarization properties of single colour centres in diamond are extremely important for applications such as in quantum cryptography where the photon polarization encodes the information, and so forth. However, if surrounding impurities in the diamond sample are illuminated by the focused excitation laser, they might be photo-ionized to generate extra electrons and even electron-acceptors. Each kind of colour centre in diamond has its unique PL properties due to the respective structure and dynamics of electrons. The dynamics of these electrons and acceptors impacts on the PL properties of adjacent colour centres, and results in fluorescence fluctuations, such as blinking, intensity jumps, and spectral changes^23^,^24^,^25^,^26^,^27^,^28^,^29^. Therefore, it is important to understand the role of the environment in polarization property of a single colour centre.

In this paper, we report the observation of a single SiV^−^ colour centre in diamond showing fluorescence intensity switching in a two-level regime at ambient conditions, which was then proved to have two switching linear polarizations. As a result of projection onto (100) surface, the angle of the polarization is 21.7°. The switching rate between the two linear polarizations was observed to increase linearly versus excitation power. Moreover, with laser excitation less than 2 mW, the SiV^−^ colour centre emitted photons of only one linear polarization. By increasing the excitation power, the second polarization state could be activated. The polarization switching phenomenon could be explained by photoionization.

## Results

### Characterization of the polarization-switching single SiV^−^ centre

**Figure 1:**
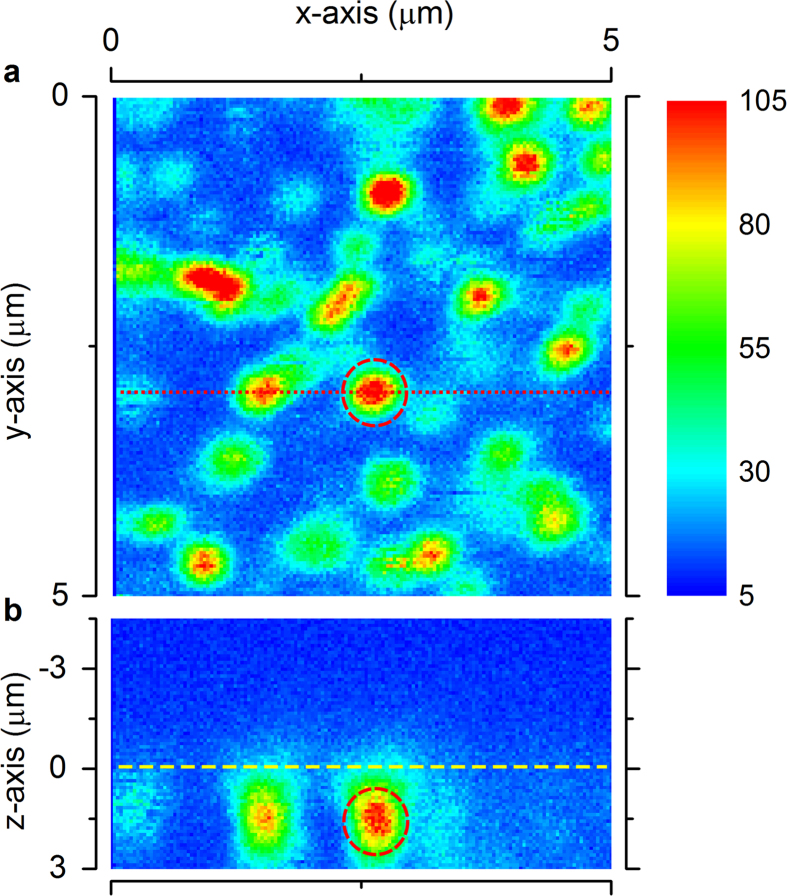
Fluorescence image of the polarization-switching single SiV^−^ centre (in red-dashed circle). **(a)** x–y surface scan; **(b)** x–z depth scan at position where a thin red dot line was marked in **a**, yellow-dashed line indicates the diamond sample surface. Colour bar embodies the PL intensity (kcounts/s).

**Figure 2:**
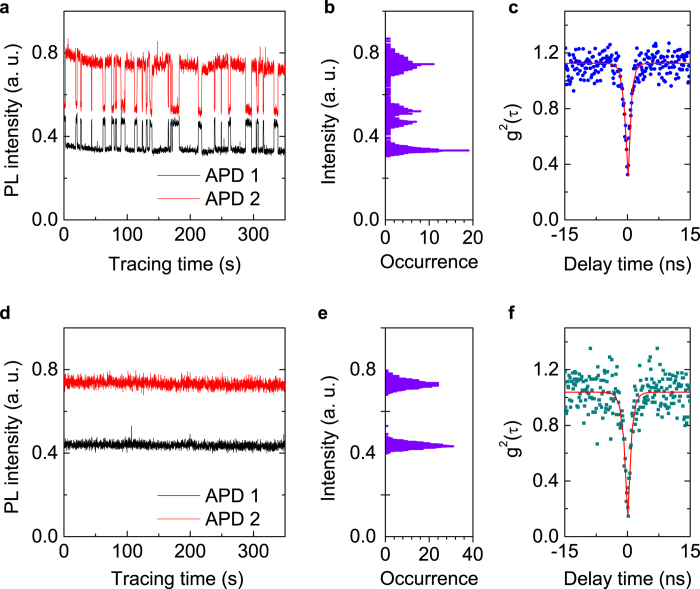
Comparison of the intensity-jumping SiV^−^ and a normal SiV^−^. **(a)** Polarization fluorescence intensity traces of the polarization-switching single SiV^−^ centre. **(b)** Fluorescence intensity occurrence histograms corresponding to **a**. **(c)** Second-order correlation function measurement of the polarization-switching single SiV^−^ centre with excitation power of 7.67 mW. **(d)** Polarization fluorescence intensity traces of a normal SiV^−^ centre. **(e)** Fluorescence intensity occurrence histograms corresponding to **d**. **(f)** Second-order correlation function measurement of the normal SiV^−^ centre with excitation power of 6.0 mW.

A diamond containing SiV^−^ colour centres prepared by ion implantation was investigated with a scanning confocal microscope. With the continuous-wave excitation laser at 532 nm, we observed single SiV^−^ colour centres distributed with a density of about 1.4/μm^2^, as shown in Fig. 1(a). Among them, an isolated SiV^−^ centre showed fluorescence polarization switching, marked with a red dashed-line circle in Fig. 1. As shown in the z-scan image of Fig. 1(b), this SiV^−^ centre is located less than 1 μm under the diamond sample surface, and as can be seen, there were no other emitters within the focal volume of the excitation laser. In the experiment, fluorescence from the SiV^−^ was resolved into two orthogonal polarizations by applying a polarized beam-splitter (PBS). (See Methods). Figure 2(a) shows the PL intensity tracing by two avalanche photo-diodes (APD) at the ports of the PBS as the SiV^−^ was excited by 21.6 mW laser. In Fig. 2(b) we histogram the PL intensities monitored by the two APDs, giving a more clear description of polarization switching. Two distinguishable peaks could be clearly observed for each polarization direction. We connected the outputs of the two APDs to the “Start” and “Stop” inputs of a time-correlated single-photon counter (Pico Harp 300) to measure the photon correlation. The photon counting rates of the two APD were adjusted to be closely balanced by the half-wave plate (HWP) before the PBS for the photon correlation measurement. A dip in photon coincidence counts to ~0.35 at zero delay appeared with excitation power of 7.67 mW [Fig. 2c], indicating a unitary photon emitter. According to the second order correlation function fitting, the characterized g^2^ (τ) time of the SiV^−^ colour centres could be deduced to be about 0.84 ns. For comparison, we tested another stable SiV^−^ colour centre nearby. As shown in Fig. 2(d–e), no switching of intensity was observed. A dip valued 0.14 at zero delay was seen in the second order correlation function measurement with excitation power of 6.0 mW in Fig. 2(f). And the characterized g^2^ (τ) time was about 0.82 ns, similar to that of the polarization switching one.

### Polarization properties

**Figure 3:**
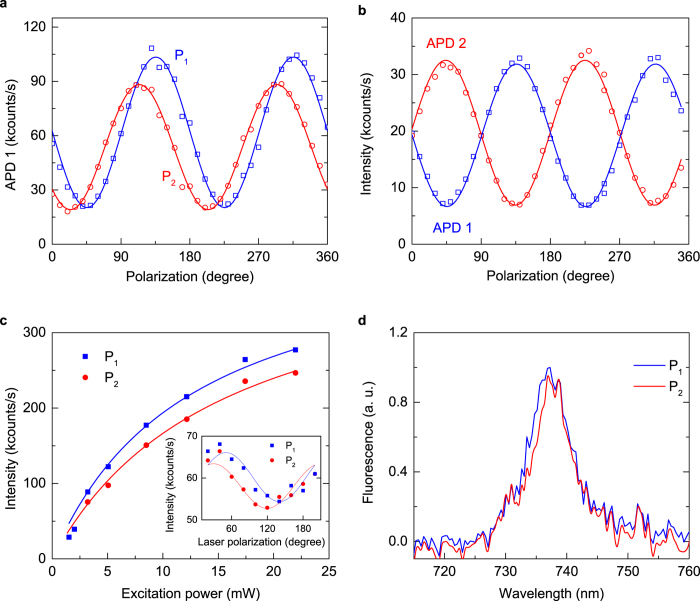
Optical properties of the two switching states. **(a)** Polarized fluorescence intensity as a function of the polarization angle at excitation power of 6.7 mW. **(b)** Fluorescence polarization at a low excitation power of 1.62 mW. **(c)** Total fluorescence intensity as a function of excitation power. Blue and red curves are fits to P_1_ and P_2_ states, respectively. Inset: fluorescence intensity of the two states versus excitation polarization. **(d)** Spectra of the polarization-switching SiV^−^ center at P_1_ state (blue solid curve) and P_2_ state (red shaded curve). The spectra were taken with the excitation power of 20 mW.

To investigate the polarization switching properties, we recorded the photon counting rates, sampling every 100 ms by each APD over 400 seconds while rotating the HWP in front of the PBS. Statistics on the photon-counting rates formed an occurrence histogram for the fluorescence intensities. There were two intensity peaks in the histogram, and we plotted the intensity vs polarization angle for each of them in Fig. 3(a). The excitation power was 6.7 mW. Fitting with a sine function, we clarified that the observed linear polarizations switched by an angle of 21.7°.

In this experiment, the polarization contrast for the two polarization states were 67% and 64%, respectively, in good agreement with the expected SiV^−^ colour centre fluoresence polarization for this diamond orientaion^14^. The fluorescence polarization of all other oberved SiV^−^ centres which do not exhibt polarization switching are along {110} with small deviations, which is consistent with Ref. 16. The SiV^−^ has a symmetric axis along {111} and the dipole moments are either perpendicular to {111} or along {111}. In our experiment, fluorescence were collected through (100) plane and the polarization contrast was lowered^30^. The 45° oriented dichromatic mirror may also slightly decrease the polarization contrast^16^. Therefore, we could conclude that the fluorescence from the SiV^−^ was switching between two linear polarization states P_1_ and P_2_.

The polarization directions of SiV^−^ were measured at different laser powers. At low excitation power, the fluorescence of the SiV^−^ showed only one polarization, which was along the same direction of P_1_ state as shown in Fig. 3(b). When the laser power was above 2 mW, the polarization switching between the P_1_ and P_2_ states occurred. With increasing the excitation power, no change on the two polarization directions was observed. Therefore, there existed an excitation laser power threshold for the polarization switching at about 2 mW.

### Excitation saturation

The PL intensity of this emitter as a function of excitation power was also investigated. The PL intensity saturation curves varied slightly for the two polarization states as shown in Fig. 3(c). The PL saturation curves were fitted by


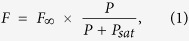


where *P*_*sat*_ is the saturation excitation power and 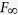 is the fluorescence counting rate in the limit of infinite excitation power. Here we obtained for the P_1_ state, *P*_*sat*_ = 13.5 mW, 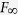 = 452 kcounts/s, and for the P_2_ state, *P*_*sat *_= 15.6 mW, 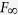 = 426 kcounts/s by fitting the saturation curves. The difference in the saturation powers could be explained by the overlap of the center’s dipole with the excitation laser’s polarization since a linear polarized laser was used in the experiment. We varied the excitation laser polarization and monitored the intensity and found that the fluorescence from the SiV^−^ showed slight dependence on the excitation laser polarization. And there was an angle difference of about 22° between the two polarization state of P_1_ and P_2_, agreeing with the polarization switching angle. Therefore, it could be concluded that the different excitation efficiency of the two states was caused by the dipole orientations with respect to the excitation laser polarization.

### PL Spectra of the two polarization states

The PL spectra of the SiV^−^ centre for the two polarization states were measured and are shown in Fig. 3(d). The ZPL peak at 738 nm provides clear evidence that the investigated defect is a SiV^−^ centre. When the SiV^−^ centre was in P_2_ state, the PL spectrum is narrower than in P_1_ state, and shifted towards to longer wavelength by about 1.6 nm. The spectra of the two states didn’t change with the excitation power. Therefore, we deduced that the spectral shift was not related to temperature change by laser illumination. Otherwise the ZPL position should show a blue shift upon cooling^25^. Additionally, there was no clear difference at the phonon-sidebands and we didn’t observe the SiV^0^ spectral peak at 946 nm which is different from the photoionization of NV centres^20^,^24^.

### Dependence of polarization switching on laser power

**Figure 4:**
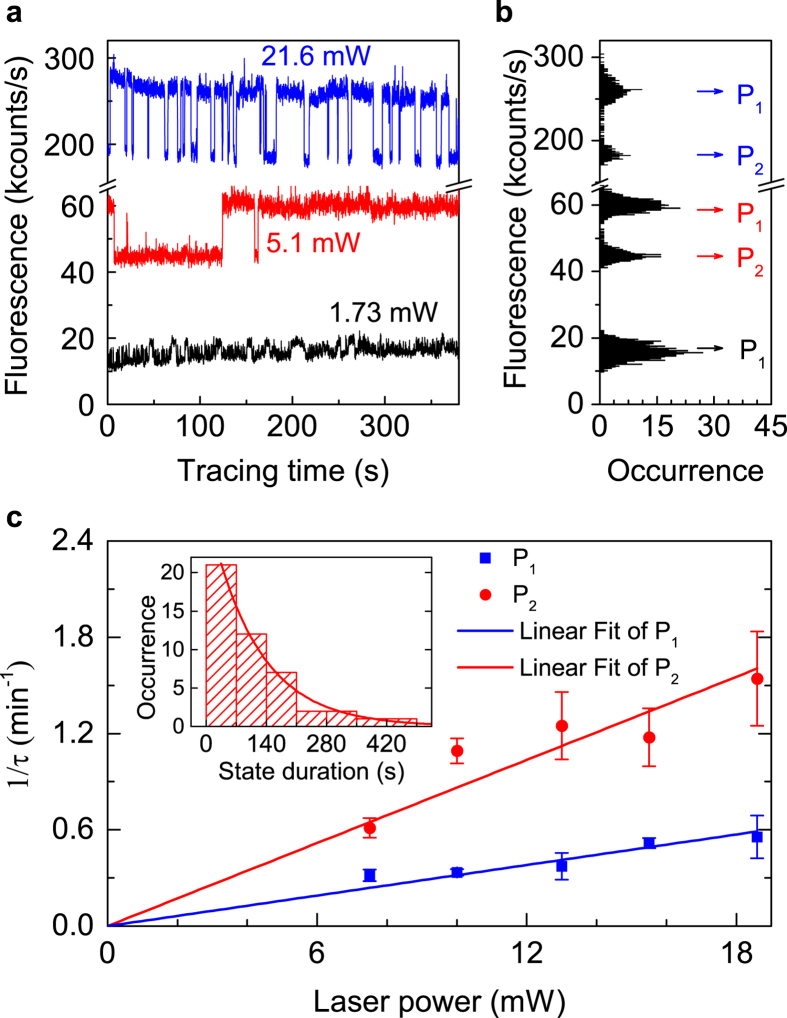
Fluorescence intensity tracing with different excitation powers. **(a)** Fluorescence intensity tracing of the SiV^−^ colour centre by APD1 at different excitation power. For the 1.73-mW power measurement, the intensity fluctuations were the same on both APDs, and not the result of polarization switching. **(b)** Histograms of fluorescence intensity corresponding to **a**, and the two switching states are labeled with arrows, respectively. **(c)** Duration time of the polarization-state τ, is dependent on excitation laser power. Inset: the statistics of P_1_ state duration time under excitation of 15.5 mW fitted according to Eq. (2).

We varied the excitation power and found that the frequency of polarization switching was dependent on the excitation laser power as shown in Fig. 4(a). According to the intensity histogram in Fig. 4(b), the state duration of P_1_ was longer than that of P_2_. With the increase of the excitation power, the polarization switched more frequently. Accordingly, the duration for the emitter to stay at either of the two polarization states was dependent on the excitation power. We analyzed the PL intensity traces at different excitation powers. With intensity tracing during 1–3 hours, about 40 “switching” events were recorded. For each excitation power, the statistics of the state duration were divided into 7–8 time periods to form a histogram, and a decay function was used to fit the histogram to obtain the characteristic time of the state duration τ,


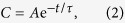


where *C* is the occurrence counts of state-duration at a certain time period, and *A* is amplitude of the function. Fig. 4(c) shows the inversion of τ as a function of excitation power, which increases linearly. In addition, at all the measured different excitation powers, the τ of P_1_ state values greater than τ of P_2_ state.

### Pre-selection of the two states

**Figure 5:**
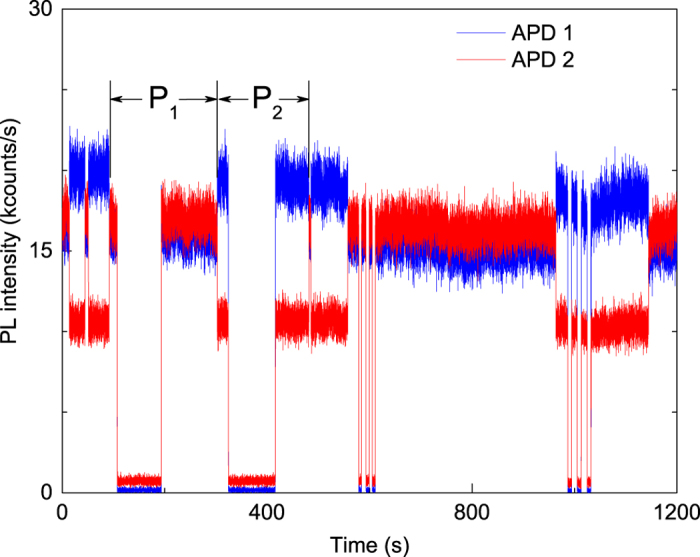
Tracing of the fluorescence intensity on the two APDs with interruptions of excitation laser.

By tracing the fluorescence intensity of the polarization switching SiV^−^ with interruption and retrieve of the excitation laser, we investigated the polarization state stability of the SiV^−^ centre under dark condition as shown in Fig. 5. The excitation laser was 20 mW to provide relatively high switching rate. We turned off the excitation laser for over 100 seconds, and when we retrieved the laser we found that the polarization direction maintained in the dark condition. Short-term interruptions of laser excitation didn’t change the polarization neither. Hence, when the excitation laser power was above the threshold of polarization switching, there was no pre-selection of the two states before an excitation laser illuminates the SiV^−^ centre.

## Discussion

Fluorescence intensity jumping of diamond colour centres has been observed and reported in Refs 26, 27, 28, 29, and ZPL shifts were observed along with fluorescence intensity jumping^26^,^27^. Such jumps might be associated with a modification of the defect configuration^26^. For example, chromium-based colour centres in diamond were observed with spectral and intensity switching but no polarization switching^27^. The phenomena were considered to be optically induced changes in the charge configuration in the vicinity of each centre, possibly related to photoionization of the defects in the diamond lattice. And Ni/Si centres were also observed with intensity jumping in a three-level regime due to the trapping or release of charges by impurity atoms or defects in the vicinity of the colour centres^28^. Surrounding defects affected by reversible changes in their charge state may also cause intensity jumping of the colour centres^29^. Therefore, the switching fluorescence properties might be a result of the photoionization of the colour centre itself or the photoionization of impurities in the vicinity positions of the colour centre which affects the quantum yield of colour centres.

Here in our observation, intensity jumps were accompanied with the spectra and polarization-switching. In our opinion, it was not due to the ionization of the SiV^−^ centre itself because SiV^−^ would not retrieve to any pre-state during quite long dark time and the spectra always presented a typical SiV^−^ peak at 738 nm and didn’t show a SiV^0^ peak, which is different with the case of photoionization of NV colour centre reported in Ref. 24. Therefore, we believe the switching fluorescence properties is associated with photoionization of impurities in the vicinity of the SiV^−^ centre. The photo-ionization of diamond impurities generates additional electron donors and stationary charge traps, which would lead to charge fluctuations in the volume of the laser focus so that the temporary local electric field surrounding SiV^−^ centres would change in the charge fluctuations. In this experiment, from time to time, the electric field altered the quantum efficiency as well as polarization properties and the spectra of the SiV^−^ centre.

In conclusion, a single SiV^−^ colour centre in bulk diamond was observed showing fluorescence polarization switching along two directions, which was also demonstrated to be a single-photon emitter by photon correlation measurement. The angle between the two polarizations projecting on the (100) plane was 21.7°. The switching rate of the two polarizations was dependent on the excitation power. Both of the two states could persist over long non-illuminating time. We attribute the whole phenomena to photoionization of unknown impurity atoms adjacent to SiV^−^ in the diamond. The experimental result benefits for a better understanding of polarization dynamics which is important for applications of SiV^−^ centres in quantum information processing and communications.

## Methods

### Sample fabrication

The diamond sample used in the experiment was a (100)-oriented diamond layer of ~15 μm thickness, grown homoepitaxially on a HPHT type Ib crystal by microwave plasma chemical vapor deposition. The grown layer was high purity diamond as estimated by confocal microscopy before ion-implantation. SiV^−^ centres were fabricated by shallow implantation of silicon atoms with 60 keV acceleration energy per ion into the sample surface followed by a subsequent anneal at 1000 °C for 30 min in 10% H_2_ forming gas.

### Confocal microscope set-up and optical measurements

**Figure 6:**
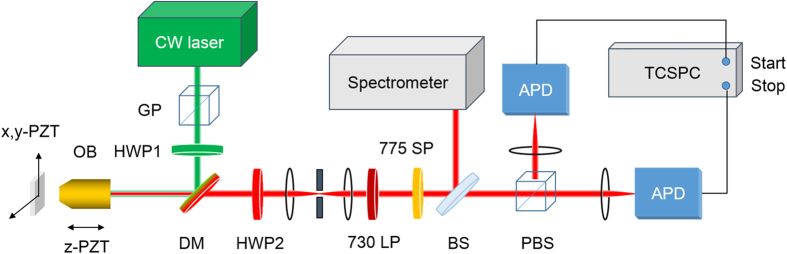
Scheme of the experiment setup. GP, Glan prism. DM, dichroic mirror reflecting excitation laser of 532 nm and passing the fluorescence longer than 540 nm. HWP1, 2, half-wave plate. OB, objective. PZT, piezoelectric ceramic transducer. 730 LP, long-pass filter cutting off at 730 nm. 775 SP, short-pass filter cutting off at 775 nm. BS, non-polarized beam-splitter. PBS, polarized beam-splitter. APDs, single-photon counting module based on avalanche photo-diode. TCSPC, time-correlated single-photon counter.

The experimental setup consisted of a homemade confocal scanning microscope combined with a spectrometer (SpectraPro-300i, Acton Research Corporation) and a time-correlated single-photon counter^31^,^32^. A scheme of the setup is presented in Fig. 6. In the confocal microscopy, an oil-immersion objective (UPlanSApo 60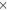/1.35 Oil, Olympus) was used to focus the linear polarized continuous-wave excitation laser at 532 nm on the surface of the diamond sample and collect the fluorescence from the colour centres at the same time. The collected fluorescence from SiV^−^ colour centres was spatially filtered by a 30-μm pinhole, and spectrally filtered by a long pass filter at 730 nm and a short pass filter at 775 nm, and then sent to a Hanbury-Brown and Twiss setup consisting of polarized beam splitter and two silicon avalanche photodiode (APD) single-photon detector (SPCM-AQR-14, Perkin Elmer). The PBS transmits horizontally polarized photons and reflects vertically polarized photons. Therefore, the horizontal and vertical components of the SiV^−^ fluorescence intensity could be measured by the two APDs simultaneously. A half-wave plate was inserted in front of the PBS in order to adjust counting rate for the two APDs. The photon counts from the APDs were acquired by a data acquisition card (NI6251, National Intruments) and a time-correlated single-photon counter (Pico Harp 300, PicoQuant).

## Additional Information

**How to cite this article**: Liu, Y. *et al.* Fluorescence Polarization Switching from a Single Silicon Vacancy Colour Centre in Diamond. *Sci. Rep.* **5**, 12244; doi: 10.1038/srep12244 (2015).

## References

[b1] Kurtsiefer C., Mayer S., Zarda P. & Weinfurter H. Stable solid-state source of single photons. Phys. Rev. Lett. 85, 290–293 (2000). 10991265 10.1103/PhysRevLett.85.290

[b2] Wu E *et al.* Narrow-band single-photon emission in the near infrared for quantum key distribution. Opt. Express 14, 1296–1303 (2005). 19503453 10.1364/oe.14.001296

[b3] Wu E *et al.* Room temperature triggered single-photon source in the near infrared. New J. Phys. 9, 434 (2007).

[b4] Liu Y. *et al.* Fabrication of nitrogen vacancy colour centres by femtosecond pulse laser illumination. Opt. Express 21, 12843 (2013). 23736503 10.1364/OE.21.012843

[b5] Aharonovich I. *et al.* Chromium single-photon emitters in diamond fabricated by ion implantation. Phys. Rev. B 81, 121201 (2010).

[b6] Wang C., Kurtsiefer C., Weinfurter H. & Burchard B. Single photon emission from SiV centres in diamond produced by ion implantation. J. Phys. B: At. Mol. Opt. Phys . 39, 37–41 (2006).

[b7] Beveratos A. *et al.* Single photon quantum cryptography. Phys. Rev. Lett. 89, 187901–187904 (2002). 12398636 10.1103/PhysRevLett.89.187901

[b8] Jacques V. *et al.* Experimental realization of Wheeler’s delayed-choice Gedanken experiment. Science 315, 966–968 (2007). 17303748 10.1126/science.1136303

[b9] Maze J. R. *et al.* Nanoscale magnetic sensing with an individual electronic spin in diamond. Nature 455, 644–647 (2008). 18833275 10.1038/nature07279

[b10] Balasubramanian G. *et al.* Nanoscale imaging magnetometry with diamond spins under ambient conditions. Nature 455, 648–651 (2008). 18833276 10.1038/nature07278

[b11] Taylor J. *et al.* High-sensitivity diamond magnetometer with nanoscale resolution. Nat. Phys . 4, 810–816 (2008).

[b12] Acosta V. *et al.* Diamonds with a high density of nitrogen-vacancy centres for magnetometry applications. Phys. Rev. B 80, 115202 (2009).

[b13] Vlasov I. *et al.* Molecular-sized fluorescent nanodiamonds. Nature Nanotechnology 9, 54–58 (2014). 10.1038/nnano.2013.255 24317283

[b14] Rogers L. J. *et al.* Electronic structure of the negatively charged silicon-vacancy centre in diamond. Phys. Rev. B 89, 235101 (2014).

[b15] Hepp C. *et al.* Electronic structure of the silicon vacancy colour centre in diamond. Phys. Rev. Lett. 112, 036405 (2014). 24484153 10.1103/PhysRevLett.112.036405

[b16] Neu E., Fischer M., Gsell S., Schreck M., & Becher C. Fluorescence and polarization spectroscopy of single silicon vacancy centres in heteroepitaxial nanodiamonds on iridium. Phys. Rev. B 84, 205211 (2011).

[b17] Rogers L. J. *et al.* Multiple intrinsically identical single-photon emitters in the solid state. Nat. Commun. 5, 4739 (2014). 25162729 10.1038/ncomms5739

[b18] Sipahigil A. *et al.* Indistinguishable photons from separated silicon-vacancy centres in diamond. Phys. Rev. Lett. 113, 113602 (2014). 25259977 10.1103/PhysRevLett.113.113602

[b19] Goss J. P., Jones R., Breuer S. J., Briddon P. R., & Öberg S. The twelve-line 1.682 eV luminescence centre in diamond and the vacancy-silicon complex. Phys. Rev. Lett. 77, 3041 (1996). 10062116 10.1103/PhysRevLett.77.3041

[b20] D’Haenens-Johanss U. F. S. *et al.* Optical properties of the neutral silicon split-vacancy centre in diamond. Phys. Rev. B 84, 245208 (2011).

[b21] Müller T. *et al.* Optical signatures of silicon-vacancy spins in diamond. Nat. Commun. 5, 4328 (2014). 24534908 10.1038/ncomms4328

[b22] Tamura S. *et al.* Array of bright silicon-vacancy centers in diamond fabricated by low-energy focused ion beam implantation. Appl. Phys. Express 7, 115201 (2014).

[b23] Dumeige Y., Treussart F., Alléaume R., Gacoin T., Roch J. F., & Grangier P. Photo-induced creation of nitrogen-related colour centres in diamond nanocrystals under femtosecond illumination. J. Lumin. 109, 61–67 (2004).

[b24] Gaebel T. *et al.* Photochromism in single nitrogen-vacancy defect in diamond. Appl. Phys. B 82, 243–246 (2006).

[b25] Neu E. *et al.* Low-temperature investigations of single silicon vacancy colour centres in diamond. New J. Phys. 15, 043005 (2013).

[b26] Siyushev P. *et al.* Low-temperature optical characterization of a near-infrared single-photon emitter in nanodiamonds. New J. Phys. 11, 113029 (2009).

[b27] Müller T. *et al.* Wide-range electrical tunability of single-photon emission from chromium-based colour centres in diamond. New J. Phys. 13, 075001 (2011).

[b28] Steinmetz D., Neu E., Meijer J., Bolse W. & Becher C. Single photon emitters based on Ni/Si related defects in single crystalline diamond. Appl. Phys. B 102, 451–458 (2011).

[b29] Monticone D. G. *et al.* Native NIR-emitting single colour centres in CVD diamond. *New J. Phys.* 16, 053005 (2014).

[b30] Fourkas J. Rapid determination of the three-dimensional orientation of single molecules. Opt. Lett. 26, 211 (2001). 18033550 10.1364/ol.26.000211

[b31] Chi Y., Chen G., Jelezko F., Wu E. & Zeng H. Enhanced photoluminescence of single-photon emitters in nanodiamonds on a gold film. IEEE Photon. Tech. Lett . 23, 374 (2011).

[b32] Chen G. *et al.* Photoluminescence enhancement dependent on the orientations of single NV centres in nanodiamonds on a gold film. IEEE J. Sel. Top. Quantum Electron. 19, 4602404 (2013).

